# Urinary bladder *Schistosoma haematobium*-related squamous cell carcinoma: a report of two fatal cases and literature review

**DOI:** 10.1186/s40794-022-00161-x

**Published:** 2022-02-15

**Authors:** Boubacar Efared, Aïchatou Balaraba Abani Bako, Boubacar Idrissa, Daouda Alhousseini, Habiba Salifou Boureima, Haboubacar Chaibou Sodé, Hassan Nouhou

**Affiliations:** 1 Faculty of Health Sciences, Abdou Moumouni University, Niamey, Niger; 2 Pathology laboratory, Niamey National Hospital, Niamey, Niger; 3 General Referral Hospital, Niamey, Niger; 4 Service of medical biology, Niamey National Hospital, Niamey, Niger; 5 Department of surgery, Niamey National Hospital, Niamey, Niger

**Keywords:** Schistosomiasis, Bladder cancer, Squamous cell carcinoma

## Abstract

**Background:**

Schistosomiasis is still a public health issue in certain areas of developing countries (especially in sub-saharan Africa). *Schistosoma haematobium* is a proven carcinogenic agent that causes mainly bladder squamous cell carcinoma. This type of cancer has characteristic epidemiological, clinical and histopathological features with poor prognosis as compared to other urinary bladder cancers not associated with this parasite.

**Cases presentation:**

We report two fatal cases of advanced-stage bladder squamous cell carcinoma associated with *Schistosoma haematobium* in a sub-saharan developing African country (Niger), illustrating the devastating complications of this tropical neglected disease. The two cases were a 38-year-old woman and a 37-year-old male. They presented with chronic pelvic pain and hematuria. The clinical and radiological work-up revealed invasive urivary bladder tumor extended to the pelvis, that was histopathologically proven to be an invasive squamous cell carcinoma associated with *Schistosoma haematobium*. The two patients died shortly after the diagnosis before chemotherapy prescription.

**Conclusion:**

Schistosoma-associated bladder squamous cell carcinoma has characteristic features with dismal prognosis. Eradication of this parasite remains the only efficient way to prevent the devastating consequences of this particular cancer.

## Introduction

Schistosomiasis is still a public health burden in certain areas of developing countries [1–3]. Schistosomiasis affects around 240 million people worldwide, and more than 90% of all cases occur in Africa. The two main schistosome species encountered in Africa are *Schistosoma mansoni* which causes intestinal and hepatic schistosomiasis and *Schistosoma haematobium*, which causes urogenital schistosomiasis [4]. There are 5 main species: *S. mansoni, S. haematobium, S. japonicum, S. intercalatum,* and *S. mekongi. S. haematobium* is responsible for chronic urogenital infections that may cause serious complications: bleeding, anemia, chronic renal failure, cancer [1, 5, 6].

Bladder cancer associated with Schistosomiasis has particular epidemiological, clinical and histopathological features [2, 7, 8]. However the mechanism by which it occurs is still a controversial issue [1, 5, 9]. Chronic interaction with the host immune system as well as association with other carcinogenic agents such as cigarettes smoking lead to the neoplastic transformation of the urinary bladder epithelium [5, 10]. In Sub-Saharan Africa the epidemiological scenario is even complex with association of many risk factors such as increasing tobacco smoking, malaria and the human immunodeficiency virus (HIV) infection [10, 11].

We report herein, two fatal cases of advanced-stage bladder squamous cell carcinoma associated with schistosomiasis from Niamey (Niger River Valley), Niger in order to point out the devastating consequences of this neglected tropical disease. It is estimated that 3.2 million people are infected with schistosomiasis in Niger [4]. Both *Schistosoma haematobium* (urogenital) and *Schistosoma mansoni* (intestinal) are endemic in Niger but the main species is *S. haematobium*, which is distributed in all regions of the country [12, 13]. Previously *S. mansoni* had a relatively marginal role; however, more recently an increase in infection has been seen in the western part of the Niger River Valley [12].

Efficient public health policies should be implemented to control the disease prevalence and its complications in poorer tropical and subtropical countries where the infection is endemic and adequate diagnostic and therapeutic tools are lacking [1].

## Cases

### Case 1

**Fig. 1:**
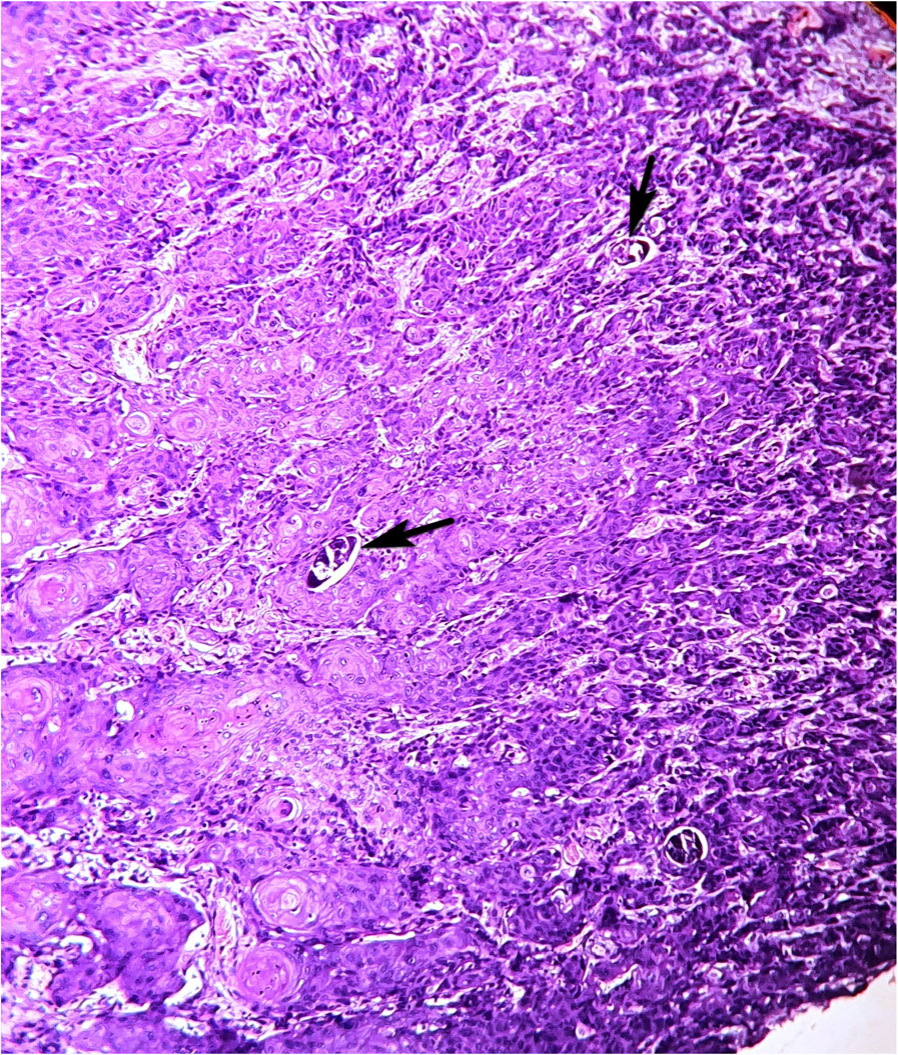
Case 1: Histological image showing a well differentiated and keratinized squamous cell carcinoma associated with *Schistosoma haematobium* calcified eggs (arrows) (hematoxylin and eosin stain × 200)

A 38-year-old woman presented with severe anemia, chronic pelvic pain, dysuria, hematuria and urinary obstruction. She is from a village in the Niger River valley where *Schistosoma haematobium* is endemic. The patient was HIV negative. The clinical examination and computed tomography-scan revealed an invasive bladder tumor. Surgeons decided to perform surgical treatment. During the procedure, they discovered a malignant bladder tumor invading the uterine cervix and the anterior vaginal wall. They decided to perform a biopsy as the tumor is beyond the bladder, thus not resectable. The histopathological analysis showed a well differentiated and keratinized squamous cell carcinoma invading the muscularis propria and the serous layer of the bladder. Tumor cells are atypical with abundant eosinophilic cytoplasm, irregular nuclei and conspicuous nucleoli with many mitoses and keratin whorls. Within the tumor, many *Schistosoma haematobium* calcified eggs are seen with their characteristic terminal spine (Fig. 1). Unfortunately the patient died weeks later before chemotherapy prescription. The patient died from severe anemia, cachexia and loss of appetite.

### Case 2

**Fig. 2:**
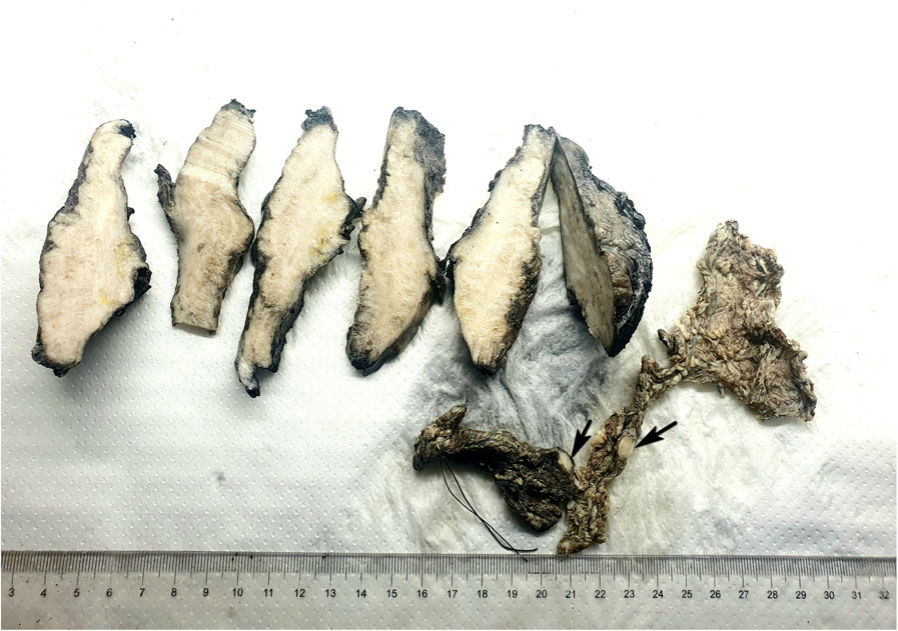
Case 2: Macroscopic resected specimens (after formalin fixation and inking) showing the partial cystectomy largely occupied by an ill-defined infiltrative whitish tumor, with 2 epiploic fragments invaded by tumoral nodules (arrows)

**Fig. 3:**
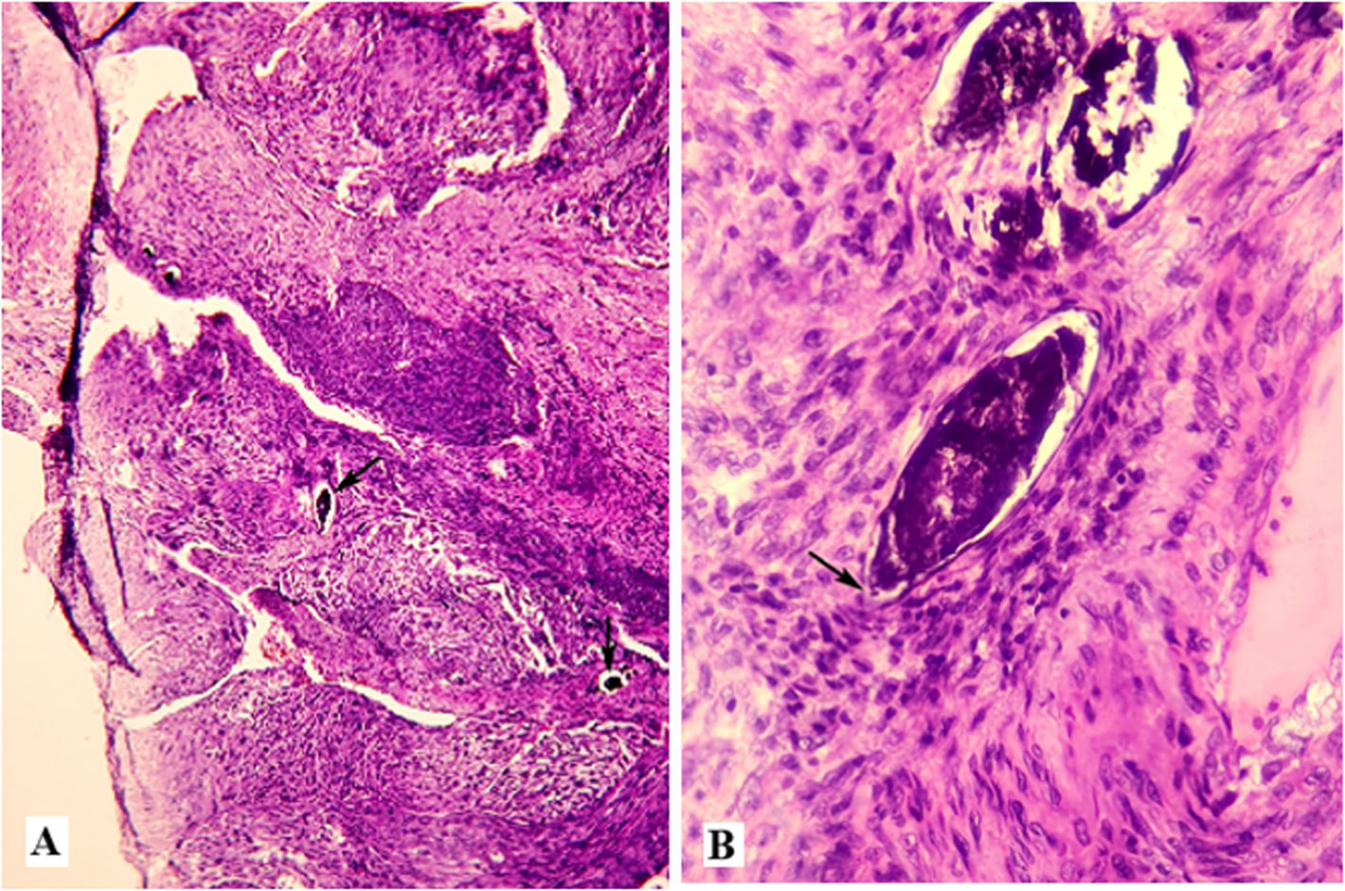
Case 2: Histological image showing a well differentiated and keratinized squamous cell carcinoma associated **A**, with *Schistosoma haematobium* calcified eggs with their characteristic terminal spine **B** (hematoxylin and eosin stain × 100)

A 37-year-old male patient was admitted in hospital (Niamey National Hospital) for chronic pelvic pain and hematuria. He is from a village in the Niger River Valley where *Schistosoma haematobium* is endemic. The patient was HIV negative. The clinical and imaging analysis revealed a localised bladder tumor. Partial cystectomy and omentectomy were performed. The gross examination of the resected specimens showed a 10 × 8 × 3 cm bladder fragment largely occupied by an ill-defined infiltrative whitish tumor, with 2 epiploic fragments of 6 to 9.5 cm in greatest dimension invaded by tumoral nodules ranging from 0.5 to 1 cm of diameter (Fig. 2). The histological analysis disclosed the diagnosis of a well-differentiated and keratinized squamous cell carcinoma invading the muscle and subserousal bladder walls with perineural invasion as well as epiploic infiltration by tumor cells. The surgical margins of the partial cystectomy were negative. Within the tumor there were many *Schistosoma haematobium* calcified eggs with their characteristic terminal spines (Figs. 3 A,B). Around the tumor squamous metaplastic epithelium was observed. The tumor was classified as pT4N0M0 (according to the American Joint Commitee on Cancer, cancer staging manual, 8th edition).

The patient died 3 weeks later before chemotherapy administration. He died from cachexia, loss of appetite with a very poor general health condition.

## Discussion

Bladder cancer associated with schistosomiasis has frequently been reported in Schistosoma endemic regions of the world especially in African countries [7, 8]. This Schistosoma-associated cancer has peculiar features: younger age, advanced-stage at diagnosis and squamous cell type histology [1, 7]. In contrast in Western countries and in non-endemic areas, the mean age of patients with bladder cancer is higher and urothelial carcinoma is the most frequent histological type (more than 90%) [7, 14, 15]. Our current cases typically illustrate these particular features of Schistosoma-associated cancer (younger patients of 37 and 38 years, with advanced-stage squamous cell carcinoma). Cases of bladder squamous cell carcinoma have been reported in Western and industrialised countries and they differ from those found in Schistosoma-endemic areas [16]. Patients are older (mean age ranging around 65 to 68 years) but with advanced-stage cancer like in Schistosoma-endemic areas [14, 15, 17–19]. Table 1 summarises the differential characteristics between the main urinary bladder carcinomas.

**Table 1:** urinary bladder main carcinomas and their differential characteristics

Characteristics	Urothelial carcinoma	Non-Schistosoma-related Squamous cell carcinoma	Schistosoma-related squamous cell carcinoma
Geographic distribution:			
- Industrialised countries	- Frequent	- Rare	- Rare
- Areas with endemic schistosomiasis	- Rare	- Rare	- Frequent
Main risk factors	Tobacco, toxic industrial chemicals	Tobacco, chronic bladder irritation (indwelling catheter, calculi)	Chronic *S. haematobium* infection, tobacco
Age (years)	Sixth-seventh decade	Sixth-seventh decade	Third-forth decade
Clinical stage at presentation	Usually limited	Usually advanced	Usually advanced
Macroscopic aspect	Polypoïd, fungating appearance	Nodular, bulky aspect	Nodular, bulky aspect
Histological differenciation	Urothelial cell phenotype with or without squamous cell differenciation	Pure squamous cell phenotype	Pure squamous cell phenotype
Histological precursors	Urothelial hyperplasia/urothelial carcinoma in situ	Squamous metaplasia	Squamous metaplasia
Radio-Chemotherapy response	Better	Poorer	Poorer
Prognosis	Better	Poorer	Poorer

The causative role of *Schistosoma heamatobium* is largely admitted and frequently proven by epidemiological studies that usually show associated parasites eggs within the tumor [7, 8]. What is still debated is the mechanism underlying the pathophysiology of bladder cancer associated with schistosomiasis [5]. Histologically a stereotypic sequence of changes is observed in animal models and in human patients: bladder urothelial hyperplasia, squamous cell metaplasia, squamous cell in situ carcinoma and invasive squamous cell carcinoma [1, 5]. In fact, our case 2 patient had metaplastic squamous epithelium around the invasive tumor, supporting the above-mentionned histological sequence of changes associated with schistosomiasis. The WHO (World Health Organisation) considers *Schistosoma heamatobium* as Group 1 carcinogen to humans (Group 1, corresponding to suspected carcinogens with the strongest evidence) [5]. The mechanisms of bladder malignant transformation by *Schistosoma heamatobium* is mainly thought to be indirect rather than direct. The eggs deposition by adult worms in tissues induces intense chronic inflammatory reaction with subsequent release of growth factors and other biochemical substances with carcinogenic effects [5, 9, 20]. Also, this chronic inflammation alter the host local immune system leading to co-infections by bacterial and viral agents that promote malignant transformation of the bladder epithelium. Our 2 cases were HIV negative, but they lived in endemic schistosomiasis areas (Niger River Valley) and they were not cigarettes smokers, so we cannot speculate about the role played by other factors in the occurrence of their bladder cancers.

All of these mechanisms act in conjunction with environmental factors (tobacco, diet, industrial products) to induce rapid progression toward invasive squamous cell bladder carcinoma [1, 5].

The prognosis of advanced stage bladder cancer is dismal and the only efficient measure is the eradication of Schistosomal parasites with all their chain of transmission by treating exposed population by praziquantel and providing them with clean water [1, 7, 17]. These policies have been implemented with success in certain countries like Egypt with a significant epidemiological outcome [7]. Unfortunately many African countries are still behind in implementing adequate measures to control schistosomiasis and will continue to register devastating complications of this diasease (bladder cancer) as illustrated by our current reported cases.

## Conclusion

Chronic infection by *Schistosoma haematobium* sometimes leads to serious complications such as bladder cancer. Schistosoma-associated bladder cancer has characteristic features: squamous cell-type carcinoma, younger age and advanced clinical stage with a poor chemotherapy response. Eradication of the parasite remains the only efficient way to prevent the devastating consequences of this parasite infection such as bladder cancer.

## Data Availability

All data of this study are included in this published article.

## Abbreviations

*S*: Schistosoma
WHO: World Health Organisation
